# Promising Noninvasive Cellular Phenotype in Prostate Cancer Cells Knockdown of Matrix Metalloproteinase 9

**DOI:** 10.1155/2013/493689

**Published:** 2013-02-06

**Authors:** Aditi Gupta, Wei Cao, Kavitha Sadashivaiah, Wantao Chen, Abraham Schneider, Meenakshi A. Chellaiah

**Affiliations:** ^1^Department of Oncology and Diagnostic Sciences, School of Dentistry, University of Maryland, Baltimore, MD 21201, USA; ^2^Laboratory of Oral Tumor Biology, Department of Oral and Maxillofacial Surgery, Shanghai Jiao Tong University School of Medicine, Shanghai 200011, China

## Abstract

Cell surface interaction of CD44 and MMP9 increases migration and invasion of PC3 cells. We show here that stable knockdown of MMP9 in PC3 cells switches CD44 isoform expression from CD44s to CD44v6 which is more glycosylated. These cells showed highly adhesive morphology with extensive cell spreading which is due to the formation of focal adhesions and well organized actin-stress fibers. MMP9 knockdown blocks invadopodia formation and matrix degradation activity as well. However, CD44 knockdown PC3 cells failed to develop focal adhesions and stress fibers; hence these cells make unstable adhesions. A part of the reason for these changes could be caused by silencing of CD44v6 as well. Immunostaining of prostate tissue microarray sections illustrated significantly lower levels of CD44v6 in adenocarcinoma than normal tissue. Our results suggest that interaction between CD44 and MMP9 is a potential mechanism of invadopodia formation. CD44v6 expression may be essential for the protection of non-invasive cellular phenotype. CD44v6 decrease may be a potential marker for prognosis and therapeutics.

## 1. Introduction

Prostate cancer is the third most common cause of death from cancer in men. Prostate cancer is a disease of extensive metastases with secondary lesions in lymph nodes, brain, bones, and sometimes in visceral organs such as the liver and lungs. Prostate cancer patients initially respond to androgen ablation therapy. However, prolonged androgen ablation therapy results in relapse and androgen independent prostate cancer progression with bone metastasis. Bone metastasis occurs in 90% of patients with advanced stage prostate cancer. The advanced stage of prostatic carcinoma eventually metastasizes to the bones in 85–100% of cases. 

Adhesion of breast and prostate cancer cells to the bone marrow endothelial cell line is directly related to the surface expression of the hyaluronic acid (HA) receptor CD44 which is a transmembrane glycoprotein [1, 2]. CD44 binds with HA through its amino-terminal conserved region [3]. CD44 functions as a protein responsible for cellular attachment to the extracellular matrix (ECM), migration, invasion, and apoptosis [1, 4–7]. The molecular mass of conserved CD44 termed CD44-standard (CD44s) is about 85–90 kDa. This is the product of transcription of exons 1–5 and 16–20. Exons 6–15 encode for separate CD44 variant isoforms from CD44v1 (not expressed in human cells) to CD44v10 [8]. The amino terminal region also contains several sites for O-linked glycosylation and attachment to chondroitin sulphate [3]. Posttranslational glycosylation of different CD44 variants produce proteins with molecular mass ranging from 80 to 200 kDa [4]. 

The biological role of the CD44 molecules is not the same in all tumors. Along with CD44s, one or multiple splice variants may be expressed in cancer cells displaying an increased tendency for expressing larger isoforms; for example, expression of CD44v8-10 in pancreatic carcinomas [9] and CD44v6 in colorectal cancer [10] and prostate cancer [11, 12]. CD44 has been suggested to play a role in the metastatic spread of prostate cancer cells [13, 14]. However, reduced and heterogeneous expression of CD44v6 was shown in six cases of primary prostate cancer by immunohistochemistry analysis [11]. The decreased expression of CD44s has also been shown to be involved in the progression of prostate cancer to a metastatic state [15]. The role of CD44 in prostate cancer development and progression remains obscure and needs further elucidation. 

CD44s surface expression and CD44s/matrix metalloproteinase 9 (MMP9) interaction on the cell surface are associated with secretion of active MMP9 and migration/invasion of PC3 cells [1]. Disruption of CD44/MMP9 interaction on the cell surface reduces migration and invasion of PC3 cells. MMP9 knockdown of PC3 cells showed reduced CD44 at cellular and surface levels [12]. An increase in the formation of invadopodia and localization of MMP9 in invadopodia may possibly increase the invasive characteristic of PC3 cells [1, 6]. The addition of a neutralizing antibody to CD44s reduced active MMP9 at the cell surface and secreted levels. Surface expression of CD44 and activation of MMP9 on the cell surface are interdependent [1, 12]. The reciprocal activities of the two proteins on the cell surface reveal an interesting situation that poses the question, “What is the biological implication of their interaction?” We hypothesize that CD44/MMP9 proteins contribute to the high metastatic property through the formation of invadopodia. 

To address this question, we generated stable PC3 cell lines deficient in MMP9 and CD44 by RNA interference knockdown method. Downregulation of MMP9 expression switches CD44 isoform expression from CD44s to CD44v6 which is more glycosylated. These cells attain the phenotype of noninvasive cells as a result of failure in the formation of invadopodia. Expression and glycosylation of CD44v6 is accompanied with extensive cell spreading and adhesion which is due to the formation of focal adhesions and stress fibers in these cells. Our data suggest that downregulation of MMP9 increases the adhesive and noninvasive phenotype in PC3 cell through the expression of CD44v6. CD44 knockdown reduces adhesive and survival properties of PC3 cells in a time-dependent manner.

## 2. Materials and Methods

### 2.1. Materials

Antibodies to GAPDH, actin, and MMP-9 were purchased from Santa Cruz Biotechnology (Santa Cruz, CA). Antibody to CD44s and Streptavidin-HRP were purchased from Cell Signaling Technology (Danvers, MA). Antibodies specific to CD44v6 and CD44v10 were purchased from R&D Systems (Minneapolis, MN), EMD Biosciences (Gibbstown, NJ) and Bender Medsystems, Inc. (Burlingame, CA). Rhodamine phalloidin and all chemicals reagents were purchased from Sigma-Aldrich (St. Louis, MO). Matched normal tissue and tumor tissue lysates made from single person were purchased from Abcam (Cambridge, MA). 

### 2.2. Cell Lines Used for Studies and Culture Conditions

We have used metastatic carcinoma-derived cell lines which include: (i) PC3, from skeletal metastases [16, 17]; (ii) LNCaP from lymph nodes [18]; and (iii) DU-145 from brain [16]. These cell lines were obtained from American Type Culture Collection (Manassas, VA). Normal prostate epithelial (HPR-1) [19, 20] and benign prostatic hyperplasic (BPH) cells [21–23] have been used as controls. We generated stable MMP9 and CD44 knockdown PC3 cells lines using respective SiRNA or ShRNA constructs as described previously [12, 24]. Stable PC3 cell lines expressing control scrambled RNAi were used as controls. MMP9 (PC3/Si) and CD44 (PC3/Si (CD44)) knockdown PC3 cells are denoted as indicated in parentheses.

Prostate cancer cell lines and benign prostatic hyperplasic control cells (BPH) were maintained in RPMI1640 (Gibco BRL, Life Technologies, Bethesda, MD) containing 5 or 10% fetal bovine serum (FBS) and 1% penicillin/streptomycin as described previously [1]. Normal prostatic epithelial cells (HPR-1) were cultured in keratinocyte (serum-free) medium supplemented with EGF (2.5 mg/500 mL), bovine pituitary extracts (25 mg/500 mL; Gibco BRL, Life Technologies, Bethesda, MD), and 1% penicillin/streptomycin [19]. 

### 2.3. Quantitative Real-Time RT-PCR Analysis

Quantitative real-time RT-PCR (qPCR) was performed using an Applied Biosystems Prism 7000 Sequence Detection System with SYBR Green PCR Master Mix (Applied Biosystems, Foster City, CA) as described previously [25]. Primer sequences and PCR product size (in parenthesis) are as follows: hCD44s (129 bp)—forward 5′ACCGACAGCACAGACAGAATC3′; reverse 5′GTTTGCTCCACCTTCTTGACTC3′; hCD44v6 (149 bp)—forward 5′GCAGCACTTCAGGAGGTTACAT3′, reverse 5′GGTAGCTGTTTCTTCCGTTGTA3′; hCD44v10 (149 bp)—forward 5′GCAGCACTTCAGGAGGTTACAT3′; reverse 5′ATGATTTGGGTCTCTTCTTCCA3′; GAPDH (132 bp)—forward 5′CTTTGGTATCGTGGAAGGACTC3′; reverse 5′GTAGAGGCAGGGATGATGTTCT3′. All reactions were prepared in triplicate and four independent sets of samples were used in each experiment.

### 2.4. Analysis of Cell Surface Expression of CD44v6 by Biotinylation and Flow Cytometry

Cells were washed with PBS and labeled with NHS-biotin according to the manufacturer's guidelines (Pierce, Rockford, IL). In conjunction with immunoprecipitation and immunoblotting analyses, the levels of surface labeled proteins were determined as described previously [1]. Flow cytometry analysis (FACs analysis) was performed essentially as described previously [26]. 

### 2.5. Migration and Invasion Assays

Wound healing and phagokinesis assays were done as described previously [26, 27]. For invasion assays, cross-linked fluorescein isothiocyanate (FITC)-conjugated gelatin matrix-coated cover slips were prepared as described [6]. To assess the formation of invadopodia and degradation of FITC-gelatin matrix, cells were cultured on FITC-gelatin-coated cover slips for 12–14 h as shown previously [6]. Cells were fixed and stained for actin with rhodamine phalloidin as described previously [27]. Gelatin matrix and actin-stained cells were viewed and photographed with a Bio-Rad confocal laser-scanning microscope. Images were stored in TIF format and processed by using Photoshop (Adobe Systems, Inc., Mountain View, CA).

### 2.6. Deglycosylation of Proteins

Deglycosylation of CD44v6 protein was done as previously described [28]. About 500 *μ*g protein was vacuum dried and resuspended in 500 *μ*L of trifluoromethanesulfonic acid (TFMSA; SIG-158534) and 1/10th to 1/2 volume of anisole (SIG-96109). The suspension was incubated for 2–6 h on ice and the reaction was stopped with cold N-ethylmorpholine (SIG-04499; 4 : 1 volume). TFMSA treatment was done in three tubes for 2, 4 and 6 h to determine the time dependent effect on complete deglycosylation. 5–10 volumes of acetone (Merck) was added to the tube and mixed well. The mix was incubated overnight at −20°C and centrifuged for 10 min at 10000 rpm to pellet protein. The pellet was dried and resuspended in SDS-containing sample buffer (100 *μ*L) prior to SDS-PAGE and immunoblotting with an antibody to CD44v6. Immunoblotting was done as previously described [27].

### 2.7. Immunostaining

Surface localization of CD44 (CD44s, CD44v6 and CD4v10) and MMP9 was determined in cells that were not permeabilized with Triton X-100. Cells fixed for 5 min with paraformaldehyde (3.7%), washed twice with cold PBS, and blocked with blocking solution were used for immunostaining with antibodies of interest as described previously [27]. Immunostained cells were viewed and photographed with a Bio-Rad confocal laser-scanning microscope. Images were stored in TIF format and processed by using Photoshop (Adobe Systems, Inc., Mountain View, CA).

### 2.8. Immunohistochemistry

Prostatic cancer and normal tissue microarray (TMA) sections with stage and grade information were bought from US Biomax, Inc. (Rockville, MD). We have used the following tissue microarray sections containing different number of cases and cores: PR242 (12 cases with 24 cores); PR481 (24 cases with 48 cores) and PR956 with metastasis in bone and alimentary wall (40 cases with 95 cores). Sections were arranged in duplicate cores per case. TMA sections were processed, stained, and analyzed essentially as described previously [24, 29]. Images were taken with an Aperio scanscope CS system (Vista, CA). Relative distribution of interested proteins in immunostained TMA sections were semiquantitatively analyzed by two other investigators. 

### 2.9. Statistical Analysis

All values presented as mean ± SEM. A value of *P* < 0.05 was considered significant. Statistical significance was determined by analysis of variance (ANOVA) with the Bonferonni corrections (Instat for IBM; Graph pad software).

## 3. Results

### 3.1. MMP9 Knockdown Increases Expression of CD44v6

**Figure 1:**
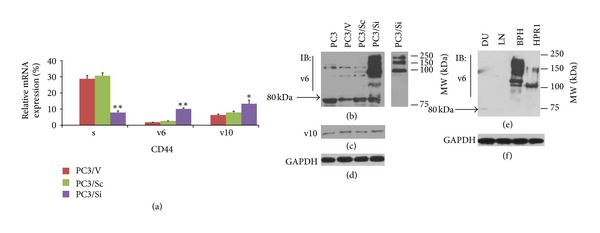
Determination of the expression of CD44s, v6, and v10 in PC3 cells knockdown of MMP9: PC3 cells transfected with vector DNA (PC3/V), scrambled nonsilencing SiRNA (PC3/Sc) and silencing SiRNA (PC3/Si) were used for real time PCR (a) and immunoblotting analyses (b–f). Untransfected PC3 cells were also used as controls. The expression levels of CD44s, CD44v6, and CD44v10 mRNA were determined by real time PCR analysis and normalized relative to GAPDH expression (a). **P < 0.001;*P < 0.01 versus indicated respective controls (PC3/V and PC3/Sc). Equal amount of protein was used for immunoblotting analysis with an antibody to CD44v6 (b and e) and CD44v10 (c). A shorter exposure blot for PC3/Si cells is shown in lane 5. The blot in (b) was stripped once and probed successively with an antibody to CD44v10 (c) and GAPDH (d). The blot in (e) was stripped and reprobed with an antibody to GAPDH. The levels of GAPDH represent the loading control for each experiment set. The results shown are representative of three or four experiments.

PC3 cells express CD44 isoforms such as CD44s, v6, and v10. MMP9 knockdown in PC3 cells (PC3/Si) reduces the expression of CD44s [12]. Here, we evaluated the expression levels of CD44v6 and v10 at RNA and protein levels in PC3/Si cells. As shown previously, MMP9 knockdown reduced the expression of CD44s at mRNA (Figure 1(a)) and protein levels (data not shown). However, real time RT PCR and immunoblotting analyses (Figures 1(a)–1(d)) displayed a significant increase in the expression of CD44v6 and v10. The increase in mRNA was found to be >3–5 fold for CD44v6 and *∼*1–1.5 fold for CD44v10 in PC3/Si cells as compared with vector (PC3/V) and a scrambled SiRNA construct (PC3/Sc) transfected PC3 cells (Figure 1(a)). 

Immunoblotting analysis showed equal levels of CD44v6 protein at molecular weight (MW) 80–85 kDa in all PC3 cells lines tested including PC3/Si (Figure 1(b)). In addition to this 80–85 kDa protein, several protein bands of CD44v6 with MW ranging between 80 and >150 kDa were observed in these cells lines. However, these bands were significantly increased in PC3/Si than PC3, PC3/V and PC3/Sc cells. An increase in the mRNA of CD44v10 (Figure 1(a)) does not appear to be translated into CD44v10 protein in PC3/Si cell line (Figure 1(c)). Equal levels of CD44v10 protein with a MW *∼*115 kDa was observed in all PC3 cell lines tested (Figure 1(c)). The correlation between increased mRNA and decreased protein levels of CD44v10 remains unclear. It is possible that CD44v10 may not have any functional significance in prostate cancer cells. 

CD44v6 expression is essentially restricted to a subset of epithelia in nonmalignant tissues [30]. Therefore, we compared the expression levels of CD44v6 in normal prostatic epithelial (HPR1) and benign prostatic hyperplasic (BPH) cells with LNCaP, DU145 prostate cancer cells (Figure 1(e)). The amount of CD44v6 in BPH and HPR1 cells (Figure 1(e)) is as good as PC3/Si cells (Figure 1(b)). Expression of CD44v6 (*∼*80 kDa) was observed in DU145 cells but at a significantly lower level (Figure 1(e), DU; indicated by an arrow). We failed to detect CD44v6 in LNCaP cells (LN). Taken together, our results demonstrate a switch in the CD44 isoform expression from CD44s to CD44v6 in PC3/Si cells. Expression of CD44v6 in PC3/Si cells as observed in HPR1 and BPH cells suggest that downregulation of MMP9 has the potential to reverse the malignant phenotype of PC3 cells.

### 3.2. MMP9 Knockdown Increases Surface Expression and Glycosylation of CD44v6

#### 3.2.1. Fluorescence Activating Cell Sorting (FACs) Analysis

**Figure 2:**
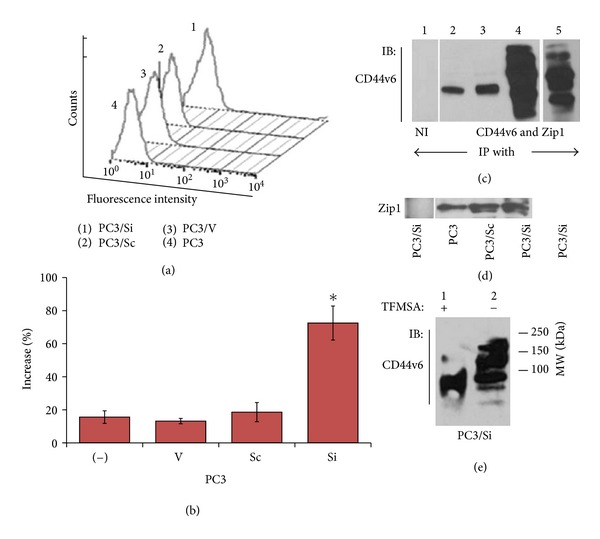
Analysis of surface expression of CD44v6 in indicated PC3 cell lines. (a) and (b), Surface expression of CD44v6 in PC3 cells knockdown of MMP9 (PC3/Si) is compared with control cell lines (PC3, PC3/V, and PC3/Sc) by FACs analysis (a) and (b). A representative histogram demonstrating the surface levels of CD44v6 in various PC3 cell lines is shown. In a typical experiments about 4–6 wells in 24 wells were used for each cell line. Statistical analysis of the mean fluorescence intensity (mean ± SEM; *n* = 3) is shown as a graph (b); *P < 0.0001 versus control cell lines. (c) and (d) Immunoblotting analysis of surface expression of CD44v6 and Zip1 proteins in indicated PC3 cell lines. Equal amount of lysate proteins (100 *μ*g) were immunoprecipitated with an antibody to CD44v6 (c) and Zip1 (d) and pulled down with streptavidin agarose. The blot was probed with a CD44v6 antibody followed by a Zip1 antibody after membrane stripping. A shorter exposure blot for PC3/Si is shown in lane 5. (e) Deglycosylation of CD44v6 with TFMSA. Total lysates (500 *μ*g protein) made from PC3/Si cells were treated with (+) and without (−) TFMSA. Immunoblotting analysis demonstrates the deglycosylated (lane 1) and non-deglycosylated (lane 2) CD44v6 (e). The results shown are representative of three different experiments.

Next, we analyzed the surface expression levels of CD44v6 in PC3/Si and control cells using flow cytometry analysis and subsequently assessed again by biotinylation procedure. A representative histogram analysis for CD44v6 is shown in Figure 2(a). A shift in the fluorescence histogram indicates an increase in the surface levels of CD44v6 in PC3/Si cells (Figure 2(a), peak 1) as compared with control cells (PC3, PC3/V, and PC3/Sc cells). Figure 2(b) is a bar graph quantitation showing a *∼*50–60% increase in PC3/Si cells. We were able to corroborate this observation in the immunoblotting analysis with lysates made from indicated cells surface labeled with NHS-biotin (Figure 2(c)). 

As shown in Figure 1, several protein bands of CD44v6 with MW ranging between 80 and >150 kDa were observed in PC3/Si cells. The level of these protein bands was significantly more in PC3/Si cells (Figure 2(c), lane 4) than control PC3 and PC3/Sc cells (lanes 2 and 3). A shorter exposure blot for PC3/Si is shown in lane 5. The blot was stripped and reprobed with an antibody to Zip1 (d). Zip1 was used as a loading control for surface proteins. It is a cell surface zinc transporter protein and was shown to express ubiquitously on the surface of PC3 cells [23]. We have previously demonstrated that osteoclasts derived from bone marrow cells express only CD44s [31]. To determine the specificity of CD44v6 antibody, we used CD44s (data not shown) and nonimmune IgG (Figure 2(c), lane 1) immunoprecipitates made from lysates of osteoclasts and PC3/Si cells, respectively. Immunoblotting analysis showed no detectable levels of CD44v6 due to nonspecific binding (Figure 2(c), lane 1) further validating the specificity of the CD44v6 antibody. 

#### 3.2.2. Deglycosylation of by TFMSA

CD44v6 contain a number of potential glycosylation sites which may explain its migration at a higher molecular weight. Trifluromethanesulphonic acid (TFMSA) was shown to deglycosylate proteins and produce predicted size peptides from cDNA [28]. Therefore, in order to determine the degree of glycosylation, total cellular lysate protein (*∼*500 *μ*g) was deglycosylated with (+) and without (−) TFMSA (Figure 2(e)). Immunoblotting analysis with an antibody to CD44v6 demonstrated that TFMSA reduces the protein size to *∼*80–85 kDa implying that Posttranslational modification (glycosylation) of CD44v6 is mediated by glycosyltransferases. The actual mechanisms of glycosylation in response to MMP9 knockdown and the glycosyltransferases which regulate glycosylation of CD44v6 have yet to be determined. 

#### 3.2.3. Immunostaining and Confocal Microscopy Analysis

**Figure 3:**
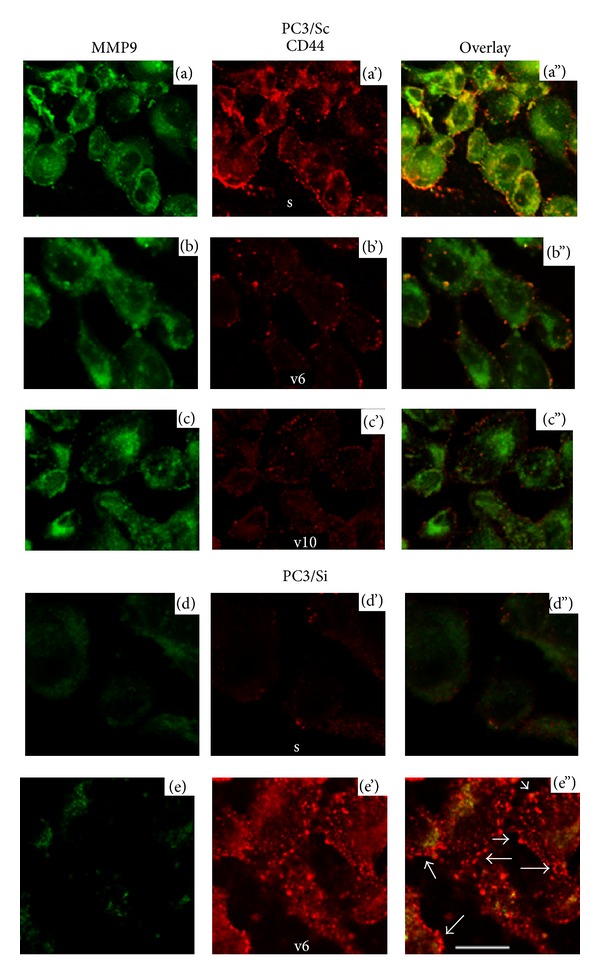
Immunostaining and confocal microscopy analysis of surface distribution of MMP9 and indicated CD44 isoforms in PC3/Sc and PC3/Si cell lines. Immunostaining was performed with antibodies to MMP9 (green) and isoforms of CD44 (s, v6, and v10; red). Distribution of both MMP9 and indicated CD44 isoforms is shown in overlay panels (a”)–(e”). Yellow color indicates colocalization of CD44s and MMP9 on the cell surface (overlay panel a”). Scale bar-50 *μ*m. The results represent one of three experiments performed.

To determine the surface distribution of indicated proteins, immunostaining was done in cells not permeablized with Triton X-100 (Figure 3). As shown previously [1], colocalization (yellow) of CD44s and MMP9 was observed on the cell surface of PC3/Sc cells (Figure 3(a”); Overlay). The expression of CD44s is reduced in PC3/Si cells (Figures 3(d') and 3(d”)), [1]. Therefore these cells (d) exhibited reduced colocalization of CD44s and MMP9 on the cell surface (d”). Surface distribution of v6 (b' and e') and v10 (c') in PC3/Sc and PC3/Si cells corroborates immunoblotting and FACs analyses shown in Figure 2. Phase contrast microscopy analysis showed that PC3/Si cells undergo a dramatic morphological alteration in cell size and shape as compared with PC3/Sc cells. PC3/Si cells are larger in size [12]. Punctate and patchy distribution of CD44v6 on the cell surface indicates that this protein is abundantly expressed by PC3/Si cells (e' and e”). Interestingly, the size of the spots appears to be larger than that observed in the control group (b'). Punctate and patchy staining indicates micro clustering of CD44v6 on the cell surface and in the periphery of plasma membrane (indicated by arrows in e”). This suggests that CD44v6 may possibly have different cellular function other than cell invasion. It is possible that CD44v6 mediated signaling may increase the adhesive nature and provide a widespread morphology to PC3/Si cells as well. Taken together, for the first time, we show here that downregulation of MMP9 is accompanied by the upregulation CD44v6 at transcriptional, translational (total and surface levels of protein) and Posttranslational (glycosylation) levels. 

### 3.3. MMP9 Knockdown Reduces Migratory and Invasive Property

#### 3.3.1. Migration Assays

**Figure 4:**
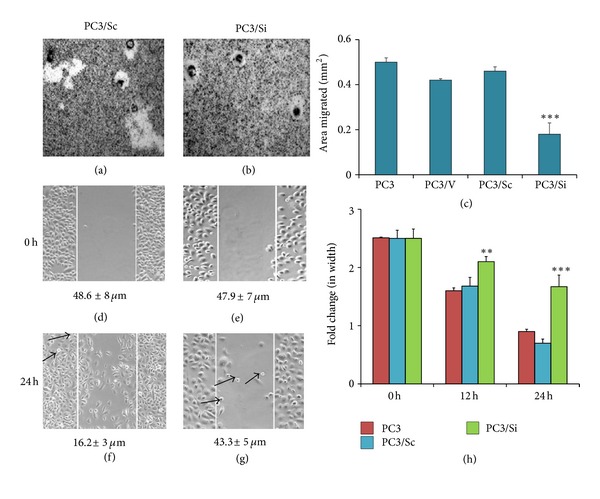
The effects of MMP9 knockdown on the migration of PC3 cells. Phase contrast micrographs of PC3/Sc and PC3/Si cells are shown (a) and (b); (d)–(g). (a)–(c) Phagokinesis assay. The area of the migratory track is seen free of the gold particles. PC3 cells were seen as black spots in the white tracks (a) and (b). Photographs were taken at the same magnification (100× in the lower-power view). The motility was evaluated by measuring areas free of gold particles and represented as mm^2^ (c). Results shown are mean ± SEM of three independent experiments. About 20 to 25 cell tracks from each experiment were measured; ***P < 0.001 versus control cells (PC3, PC3/V, and PC3/Sc). (d)–(h) Wound closure assay. A representative wound closure assay before (d) and (e) and after (f) and (g) migration of PC3/Sc (d) and (f) and PC3/Si (e) and (g) is shown. Arrows in (f) and (g) point to dead floating cells. Data showed at the bottom of each panel (in  *μ*m) represent mean ± SEM of an experiment performed in triplicates at 24 h. Statistical analysis (mean ± SEM) of three experiments performed in triplicates at 12 and 24 h is provided as a graph (h). A significant decrease in the migration and wound closure was observed at 12 and 24 h in PC3 cells knockdown of MMP9 (PC3/Si) as compared with indicated control cells. **P < 0.01, ***P < 0.001 versus control cells (PC3/Sc and PC3/V).

**Figure 5:**
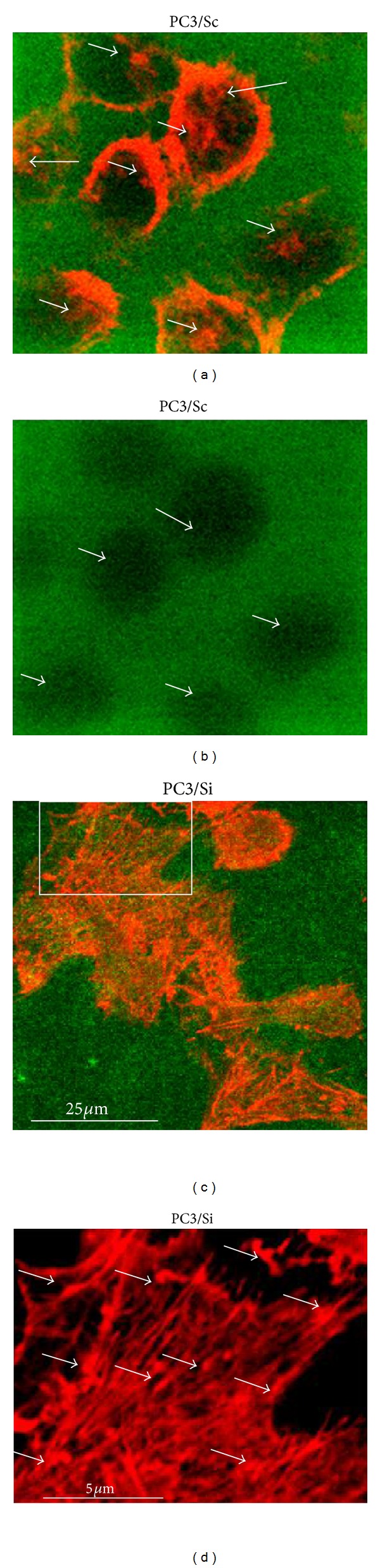
Determination of invasion property of PC3/Si and PC3/Sc cells using gelatin degradation assay. The ability of PC3 cells knockdown of MMP9 to degrade the gelatin matrix was determined by culturing PC3 cells (PC3/Sc and PC3/Si) on a FITC-conjugated gelatin matrix (green). Cells were found within the degraded matrix (a). Actin staining is shown in red (a), (c), and (d). Invadopodia are indicated by arrow heads (a). Areas of degradation of the matrix are indicated by green arrows (b). The rectangular field in (c) is enlarged in (d). Focal adhesions are indicated by arrows (d). These results represent one of several experiments performed.

Having established that MMP9 knockdown increases the expression of CD44v6 in PC3 cells, we next examined the functional consequence of this change in cell migration (Figure 4) and invasion (Figure 5) assays. Cell migration was assessed by phagokinesis (a and b) and wound healing (d–h) assays. In phagokinesis assay, PC3/Si cells displayed a significant decrease in migration (Figures 4(b) and 4(c)) as compared with PC3/Sc cells (a and c). Similar observations were made in the wound healing assay. A decrease in the wound size from 48.6 ± 8 *μ*m at 0 h (Figure 4(d)) to 16.2 ± 3 *μ*m at 24 h (Figure 4(f)) was observed in PC3/Sc cells. However, the wound size was 47.9±7 *μ*m at 0 h (Figure 4(e)) and 43.3 ± 5 *μ*m at 24 h (Figure 4(g)) in PC3/Si cells. Statistical analysis is provided as a graph at 0 h, 12 h, and 24 h (Figure 4(h)). Wound healing is comparable in PC3 and PC3/Sc cells. These cells move toward the wound and the wound area decreased over time. However, MMP9 knockdown reduces or delayed wound closure significantly. The defect in migration is reflected in the morphology and size of PC3/Si cells (Figure 4(g); [12]).

#### 3.3.2. Invasion Assays

Next, we proceeded to check the invasive property of PC3/Si cells using gelatin degradation assay as shown previously [12]). Cells were stained with rhodamine phalloidin for actin (red) and analyzed by a confocal laser scanning microscopy (Figure 5). PC3/Sc cells displayed actin staining in several distinct invadopodia-like structures (indicated by arrow heads in Figure 5(a)). Degradation of FITC-conjugated gelatin matrix (green) was observed (Figure 5(b), indicated by green arrows) and PC3/Sc cells were found within the excavated matrix (Figure 5(a)). However, PC3/Si cells failed to demonstrate matrix degradation (c). A discrete reorganization of actin cytoskeleton with the formation stress fibers and focal adhesions was observed in PC3/Si cells (Figures 5(c) and 5(d); indicated by arrows). Consistent with our observations in gelatin degradation invasion assay, MMP9 knockdown significantly reduced 3D extracellular matrix degradation and 3D invasion in an OrisTM Cell Invasion & Detection Assay system containing 3D surface (see Figure S1 avaliable online at http://dx.doi.org/10.1155/2013/493689). These results confirmed the dependence of prostate cancer cells migration and invasion on MMP9 activity and the formation of invadopodia.

### 3.4. CD44 Knockdown Reduces Adhesive Property of PC3 Cells

**Figure 6:**
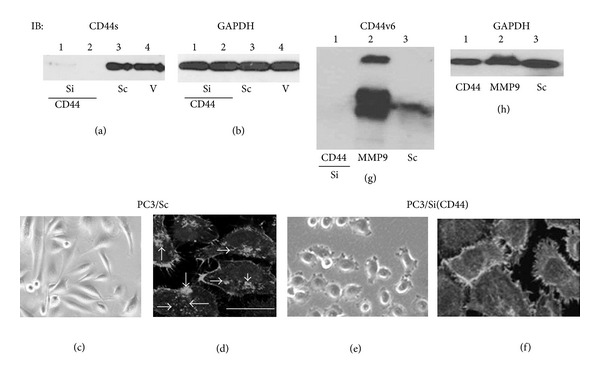
Analysis of the effects of CD44 knockdown on the expression of CD44v6, cell morphology, and actin distribution. (a), (b), (g), and (h) The effect of ShRNA to CD44 on the cellular levels of CD44s protein was determined by immunoblotting analysis. The expression levels of CD44s are shown in different PC3 cell lines by immunoblotting analysis (a). Individual clones were isolated from cells transfected with a construct corresponding to 492 bp ((a), lane 1) and 801 bp (lane 2). Cells transfected with a nonsilencing scrambled ShRNA construct (Sc; lane 3) and vector DNA (V; lane 4) were used as controls. PC3 cells knockdown of CD44s ((g), lane 1) and MMP9 (lane 2) as well as a control PC3/Sc cells (lane 3) were used for immunoblotting analysis with an antibody to CD44v6. Total cellular lysates (*∼*50 *μ*g) were used for immunoblotting analyses (a), (b), (g), and (h). Equal loading of protein was verified by immunoblotting with an antibody GAPDH (b) and (h). The experiment was repeated three times with similar results. (c)–(f) The morphology of PC3/Sc and PC3/Si (CD44) cells was determined by phase contrast microscopy (400x; (c) and (e)). Cells were stained with rhodamine phalloidin to examine the distribution of actin in PC3/Sc (d) and PC3/Si (CD44) (f) cells. Staining of actin is shown in gray scale (d) and (f). Arrows point to invadopodia in PC3/Sc cells (d). The results shown are representative of three independent experiments. Bar: 50 *μ*m.

Since MMP9 and CD44s has interdependent role in the invasion of PC3 cells, the next logical step is to understand whether knockdown of CD44 in general will have any impact on the organization of invadopodia in PC3 cells. Four different silencing and one control scramble ShRNA constructs for the CD44s cDNA sequences (Genbank-NM_000610.3; encodes the longest isoform) were made to knockdown CD44 in PC3 cells as described previously [24]. PC3 cells stably transfected with vector DNA and a scrambled nonsilencing ShRNA construct were used as controls. Several individual clones (*∼*15–20) were characterized for each construct and tested the expression levels of CD44s. A significant decrease in CD44s was observed in the clonal isolates of PC3 cells transfected with silencing ShRNA constructs corresponding to nucleotide sequences 492 and 801 bp [24]. Among the individual clones tested, we chose clones which demonstrated very minimal or no expression of CD44s for further studies (Figure 6(a), lanes 1 and 2). These cells failed to exhibit the basal level expression of CD44v6 as well (data not shown). 

The morphological changes in PC3 cells by knockdown of CD44 (PC3/Si (CD44) were assessed by phase contrast microscopy. The morphology of PC3 cells is shown in Figure 6(c). PC3/Si (CD44) cells were smaller in size and membrane bleb-like projections were seen at the periphery after the cells were adhered to cell culture dishes for 24 h (Figure 6(e)). These changes in the morphology match up with the reorganization of actin filaments into retraction of cell protrusions with the formation of numerous cytoplasmic processes or microvilli-like structures at the periphery. Neither invadopodia nor stress fibers/focal adhesions were observed in these cells (Figure 6(f)). Retraction of cell protrusions and the formation of cytoplasmic processes could be a sign of rounding up of cells as a result of reduced CD44s signaling. This may be one of the characteristic features of cell detachment. Therefore, cells were cultured for 72 h and viewed under phase contrast microscope. Cells rounded up and detached at 48 h and 72 h in a time dependent manner. Only a few cells were attached to cell culture dishes at 72 h. Cell viability is reduced after 5 days in culture (data not shown). We present evidence that CD44 signaling can control invadopodia formation and cell adhesion in PC3 cells. Knockdown of CD44 in general has the potential to reduce the invasive and survival property of PC3 cells [32]. 

### 3.5. Expression of CD44v6 Is Low in Prostatic Adenocarcinoma in Tissue Microarray Sections

**Figure 7:**
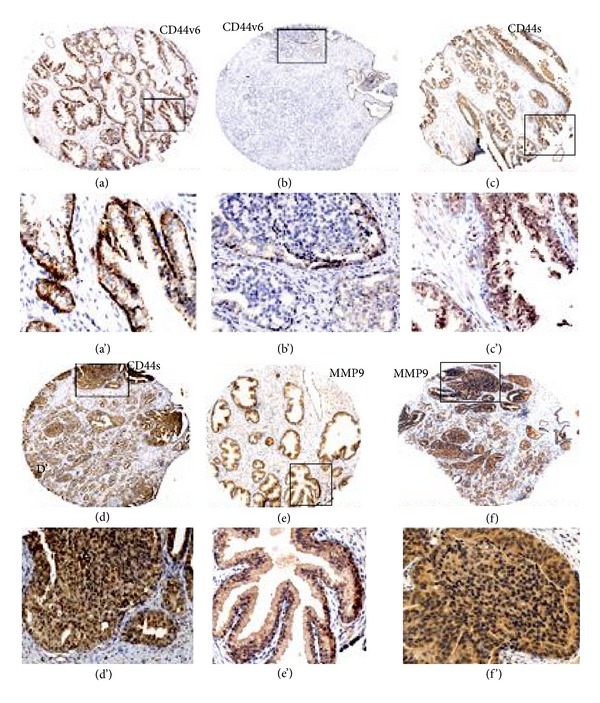
Immunohistochemical detection of CD44v6 (a) and (b), CD44s (c) and (d), and MMP9 (e) and (f) in normal (a), (c), and (e) and adenocarcinoma (grade 2-3; (b), (d), and (f)) sections of representative prostate tissues from a tissue microarray: immunostained sections (brown) with indicated antibody above were counterstained with hematoxylin stain (blue). Note the intense immunopositivity of basal epithelial membrane of epithelial cells in normal gland (indicated by arrows in (a')). Magnification is 50× in (a)–(f). Location of the high magnification (200×) regions shown in (a')–(f') is indicated by a rectangle field in (a)–(f). The staining was repeated thrice with similar results.

In order to confirm the expression levels of CD44v6 in multiple prostate cancer tissue samples from autopsy, we carried out immunohistochemistry analyses in prostate cancer tissue microarray (TMA) sections containing matched normal adjacent tissue (Figure 7). Number of cases and cores in the TMA are indicated in Materials and Methods. A representative tissue microarray panel containing sections of normal and tumor tissue (24 cores) and the cores which are selected to show at higher magnification are indicated by a rectangular field in Figure S2 (additional file). Also, semiquantitative analysis distribution of CD44v6 in normal prostatic tissue, prostatic adenocarcinoma (stage 3 and 4), and metastatic adenocarcinoma in bone is shown in Figure S3 (additional file). Medium (a–f) and higher (a'–f') power view of indicated core in Figure S2 are shown for the specificity of staining in Figure 7. 

Normal prostate tissue showed positive staining for CD44s and MMP9 in the cytoplasm and nuclei of luminal epithelial and basal cells (Figures 7(c) and 7(e)). Prostatic adenocarcinoma sections showed multiple small foci of tumor cells (d and f). One of the foci is showed in higher magnification (d' and f'). Overexpression of CD44s (d and d') and MMP9 (f and f') was clearly observed in tumor cells. CD44v6 expression is more in normal tissue (a) than in tumor tissue (b). High power view (a') demonstrated intense staining in the basal cells and basolateral plasma membrane (indicated by arrows in a') of luminal epithelial cells. Prostatic adenocarcinoma at stages 1–4 showed weak reactivity for CD44v6 (b and b'). The foci of tumor cells in the same core or tissue section which demonstrated intense staining for CD44s (d') and MMP9 (f') displayed minimal staining for CD44v6 at the periphery in the monolayer cells (b'). No staining was observed in the tumor cells found within the foci (b'). Similarly, prostatic adenocarcinoma at stages 1–4 as well as prostate cancer metastasis to bone and abdominal wall showed weak reactivity for CD44v6 (Figure S3 in additional file). Taken together, these observations imply that there is a switch in the expression of CD44v6 to CD44s occurs during metastatic process. We suggest here that downregulation of CD44v6 is related and perceived to be important in the prognosis and progression of prostate cancer. Expression of CD44s may serve as molecular marker for invasiveness. 

## 4. Discussion

The aim of this study is to understand the underlying molecular mechanisms regulating invasion in prostate cancer cells. Highly invasive cancer cells expressing invadopodia degrade and enter the matrix produced by cells like fibroblasts [33–35]. Normal cells neither form invadopodia nor degrade the ECM [36]. We have shown here and previously that localization of MMP9 in the invadopodia of PC3 cells regulates the invasion into the matrix [6]. To elucidate the possible roles of MMP9 in prostate cancer cell invasion/migration processes, we generated PC3 cells knockdown of MMP9 (PC3/Si). PC3/Si cells adhered and spread well in culture [12]. Invasion analyses revealed that PC3/Si cells are noninvasive in nature which is certainly due to failure in the formation of invadopodia. 

CD44 expression has been associated with aggressive behavior of various tumor cells [13, 37, 38]. Src, Rho, cortactin, WASP, and Arp2/3 proteins are involved in the formation of invadopodia [6, 33, 39, 40]. Failure to form invadopodia in PC3 cells treated with a WASP peptide suggests that actin polymerization and formation of invadopodia involves the WASP-Arp2/3 complex [6]. Several of these important proteins which induce invadopodia formation (e.g., cortactin, Src, Rho, and WASP) were shown to interact with CD44 [41, 42]. PC3/Si cells are noninvasive in nature which is not only due to failure in the formation of invadopodia but also due to reduced expression of CD44s. However, downregulation of MMP9 expression in PC3 cells switches CD44 isoform expression from CD44s to CD44v6 which is more glycosylated. This is accompanied with the formation of focal adhesions and stress fibers in PC3/Si cells. Focal adhesions have been shown to have both adhesive and invasive properties in pancreatic cancer cells [43]. An increase in the expression and glycosylation of CD44v6 isoform in PC3/Si cells possibly suggest a role for CD44v6 signaling in the formation of focal adhesions and stress fibers. CD44 isoforms may have unique signaling properties. It seems that the biological role of CD44 molecules is not the same in all cell types or tumors.

The expression of different CD44 isoforms has been correlated with the human tumor progression (rev. in [44]). In addition to a strong expression of CD44s, variant isoforms of CD44 (CD44v) were observed predominantly in aggressive lymphoma. This has been associated with a shorter overall survival of patients [45]. In a rat metastasis model, v6 isoform is causally involved in lung metastasis formation [44]. CD44v6 isoform appears to be the major importance for tumor dissemination in human non-Hodgkin's lymphoma, colon carcinoma and mammary carcinoma [46–49]. Downregulation of MMP9 has an impact on the expression and surface levels of CD44s. However, the intriguing observation is the expression and glycosylation of CD44v6 in PC3/Si cells corresponds with the benign (BPH) and normal prostatic (HPR1) epithelial cells. As shown by others in cases of carcinoma [50], we have shown here that the expression of CD44v6 is down regulated in prostate cancer tissue. Expression of CD44 variant isoforms containing sequences are tightly regulated and restricted to epithelial cells [45, 51, 52]. Expression of CD44v6 in HPR1 and BPH cells corroborate this observation. It is possible that PC3/Si cells may assume a noninvasive epithelial-like phenotype which may trigger the expression of CD44v6. It suggests that CD44 isoforms can exert different molecular mechanisms and functions depend on the situation. Thus, unlike what is observed in other tumors, CD44v6 appears to be inversely positive with tumor progression in prostate cancer.

The role of CD44 isoforms in prostate cancer development and progression needs further elucidation. Evidence of CD44 involved in prostate cancer is conflicting as some have shown increased expression of CD44s and CD44 variants to be associated with poor prognosis. However, others found a reduction of CD44 variants in tumors of patients with advanced carcinoma. With the use of PC3/Si and PC3/CD44s cell lines, we have provided evidence that modulation of expression of CD44s has an impact on the invasion and migration of prostate cancer cells. Little is known about the prognostic value of the expression of variant isoforms of CD44. Expression of CD44v6 may possibly an independent predictor of survival in prostate cancer. As suggested by others [53], our results support the association of expression of CD44v6 with prolonged disease-free with possibly better-survival significance. We show here for the first time that MMP9 knockdown induces noninvasive cellular phenotype in prostate cancer cells. 

We have previously demonstrated that CD44s regulates cell survival in PC3 cells [7]. One would expect a decrease in cell viability as CD44s expression is considerably reduced in PC3/Si cells. However, an increase in the expression of CD44v6 independent of MMP9/CD44s regulation compensates this mechanism and supports cell viability. CD44v6 expression inversely correlates with pathologic stage and disease progression and positively correlates with PSA-free survival [54]. We have begun studies designed to determine the specific roles of MMP9 and CD44s in tumor progression. How does this switch “CD44s to CD44v6” in PC3 cells improve survival and what is/are the mechanism(s) tied to this switch needs further elucidation. 

CD44 variant isoforms contain new oligosaccharide attachment sites which provide functionally distinct and significant glycosylation changes [55, 56]. Glycosylation of CD44v6 is suppressed in PC3 cells although its expression was observed at transcriptional and translational levels. Differential regulation of CD44v6 seems to occur during normal and nonmalignant state of cells. Glycosylation of CD44v6 may induce the formation of actin stress fibers and focal adhesions. These events can occur only upon the binding of CD44v6 with appropriate ligand. 

CD44 variant isoforms v6 and v7 have been shown to confer binding properties with multiple glycosaminoglycans (GAGs) when expressed on the cell surface [57]. CD44 binding with GAGs was suggested to have several functional consequences such as, (a) affinity to ECM to enhance cell-matrix interactions; (b) trigger signal transduction mechanism; (c) present growth factor to its receptor [57]. CD44 variants have also been shown to function as a coreceptor for the activation of growth-promoting tumor receptor tyrosine kinases [58, 59]. As suggested by others, it is possible that variant isoform may either mediate binding to new ligands, or modulate the function of domains expressed on all CD44 proteins, such as the hyaluronic-acid-binding domain. Further understanding of the GAG and ligand binding activities of CD44v6 and its role in the formation of focal adhesions will provide insight into the functions of CD44s and CD44v6 in the metastasis of tumor cells. Further biochemical characterization of how CD44v6 signaling is orchestrated spatiotemporally and complexes of proteins involved in the distinctively different adhesion regimes are in progress. Identification of the ligand that is involved in the signaling events by CD44v6 may provide a clue to develop new strategies to inhibit invasion. 

## 5. Conclusions

 The spread of cancer cells to distant sites in the body is the major cause of cancer patients' death. PC3 cells knockdown of MMP9 or CD44s fail to degrade the matrix due to the absence of invadopodia. Cells knockdown of these proteins highlights the importance of these proteins in prostate cancer invasion and migration. CD44s induced signaling events are associated with the highly metastatic property of androgen independent prostate cancer cells. PC3/Si (CD44) cells lost their ability to attach to plates due to failure in the formation of focal adhesions. Loss of invasion property in these cells is due to failure in the formation of invadopodia. Glycosylation of CD44v6 may provide a firm adhesive phenotype with the formation of stress fiber and focal adhesions. A switch from CD44 variant isoforms to CD44s can influence the metastatic property of tumor cells. Downregulation of CD44v6 signaling correlates with invasion and metastasis processes via gain of function of CD44s in androgen-independent prostate cancer cells. 

## Supplementary Material

Figure S1: Determination of migration/invasion property of indicted PC3 cells by the Oris cell invasion assay (3D assay). Images were taken by use of an inverted Nikon microscope (Eclipse TE200) with 4X (A-C), 10X (A'-C') and 40X (A”-C”) objectives. Representative images of pre-migration/invasion zones at 0h and migrated/invaded zones after 24h (A'-C') are shown. Invaded regions are shown at higher magnification in A”-C”. Cells moved through the basement membrane extract (BME) (A'-C') and invaded into the BME (A” and B”). PC3 cells knock down of MMP9 displayed a significant decrease in migration/invasion (C' and C”). Cell migration/invasion into detection zone (towards center) was represented as percent invasion at the bottom of each panel. The results shown are representative of three independent experiments.
Figure S2: Prostate cancer and normal tissue microarray (TMA). Prostate adenocarcinoma at different stages and normal tissue cores (12 cases in duplicates) are stained with indicated antibody (I-III) and non-immune IgG (IV) are shown. The immunostained cores which are selected to show at higher magnification in Figure 8 of the main document are indicated by a rectangular field above. TMA containing 24 and 40cases were also used for this analysis. Relative distribution of indicated proteins in immunostained TMA sections were semi-quantitatively analyzed by two other investigators and provided as graph in the main document.
Figure S3: Immunohistochemical detection of CD44v6 in normal prostatic tissue, prostatic adenocarcinoma (stage 3 and 4) and metastatic adenocarcinoma. A-F and A'-F': Prostate adenocarcinoma tissue microarray with duplicated cores of 36 cancer and three cases of metastasis to bone and one in abdominal wall was immunostained with an antibody to CD44v6. In the 36 cancer duplicate cores, 8 of which have matched normal adjacent tissue (Cat. No. PR956; BioMax, Inc). Representative tissue sections obtained from A-F': Immunohistochemical detection of CD44v6 in normal tissue (A and A'), adenocarcinoma at stage 3 and 4 (B, B', C and C') and metastatic cancer in abdominal wall (D and D') and bone (E, E', F and F') is shown. NT represents normal tissue. Magnification is X50 in A-F. Location of the high magnification (X200) regions shown in A'-F' is indicated by a rectangle field in A-F. G: Semi-quantitative analysis of distribution of CD44v6 in normal prostatic luminal epithelial cells in prostatic tissue (NM-LEC), prostatic adenocarcinoma (ADCA; stage 3 and 4) and metastatic adenocarcinoma in bone (METs). Expression of CD44v6 was measured semi-quantitatively via using the intensity of the immunoreactivity of CD44v6. It was calculated using the 0 to 4 tiered scale as shown in Figure S3G. The percentage was calculated based on the staining intensity of cells to CD44v6 protein in 4-tiered scale as shown in the figure G (see description below). ‘n' indicates number of cores analyzed. The staining was repeated thrice with similar results.

## Abbreviations

CD44s: Soluble or Standard CD44
FBS: Fetal bovine serum
GAPDH: Glyceraldehydes-3-phosphate dehydrogenase
RT-PCR: Reverse transcriptase PCR
TMA: Tissue microarray
IP: Immunoprecipitation
IB: Immunoblot
MMP9: Matrix metalloproteinase-9
PC3/Si: PC3 cell lines knockdown of MMP9
TFMSA: Trifluromethanesulphonic acid
CD44v: Variant isoforms of CD44
GAGs: Glycosaminoglycans
BME: Basement membrane extract.
