# Supplementary material for: Promising Noninvasive Cellular Phenotype in Prostate Cancer Cells Knockdown of Matrix Metalloproteinase 9
Source: ScientificWorldJournal. 2013 Feb 6;2013:493689. doi: 10.1155/2013/493689 (PMC3580924; doi:10.1155/2013/493689)
Supplement: Supplementary file 1 — Figure S1: Determination of migration/invasion property of indicted PC3 cells by the Oris cell invasion assay (3D assay). Images were taken by use of an inverted Nikon microscope (Eclipse TE200) with 4X (A-C), 10X (A'-C') and 40X (A”-C”) objectives. Representative images of pre-migration/invasion zones at 0h and migrated/invaded zones after 24h (A'-C') are shown. Invaded regions are shown at higher magnification in A”-C”. Cells moved through the basement membrane extract (BME) (A'-C') and invaded into the BME (A” and B”). PC3 cells knock down of MMP9 displayed a significant decrease in migration/invasion (C' and C”). Cell migration/invasion into detection zone (towards center) was represented as percent invasion at the bottom of each panel. The results shown are representative of three independent experiments. Figure S2: Prostate cancer and normal tissue microarray (TMA). Prostate adenocarcinoma at different stages and normal tissue cores (12 cases in duplicates) are stained with indicated antibody (I-III) and non-immune IgG (IV) are shown. The immunostained cores which are selected to show at higher magnification in Figure 8 of the main document are indicated by a rectangular field above. TMA containing 24 and 40cases were also used for this analysis. Relative distribution of indicated proteins in immunostained TMA sections were semi-quantitatively analyzed by two other investigators and provided as graph in the main document. Figure S3: Immunohistochemical detection of CD44v6 in normal prostatic tissue, prostatic adenocarcinoma (stage 3 and 4) and metastatic adenocarcinoma. A-F and A'-F': Prostate adenocarcinoma tissue microarray with duplicated cores of 36 cancer and three cases of metastasis to bone and one in abdominal wall was immunostained with an antibody to CD44v6. In the 36 cancer duplicate cores, 8 of which have matched normal adjacent tissue (Cat. No. PR956; BioMax, Inc). Representative tissue sections obtained from A-F': Immunohistochemical detection of [file 493689.f1.pdf]

SUPPLEMENTARY FILE

METHOD

Oris cell invasion & detection assay kit: This ssay was performed according to manufacturer's instructions (Platypus Technologies, LLC, Madison, WI). Results for this assay are provided in the supplemental section (Figure S1). Briefly, cells (~$5X10^4$ cells / well) were seeded on the Oris™ BME (Basement Membrane Extract) coated microplate with the cell seeding stoppers and allowed to adhere for 24h. After 24h, the stopper was removed using the tool provided in the kit. Cells were rinsed gently and incubated for 12-30h at 37^0C in RPMI 1640 containing 0.5% FBS. Mitomycin (5 µg/ml; Sigma) was added to the medium to inhibit proliferation. Cells migrated into the cell-free area and invaded into BME were photographed using a digital spot camera attached to an inverted Nikon phase contrast microscope at 12 and 24h. The assay was performed in duplicates and repeated thrice.

RESULTS

Figure S1

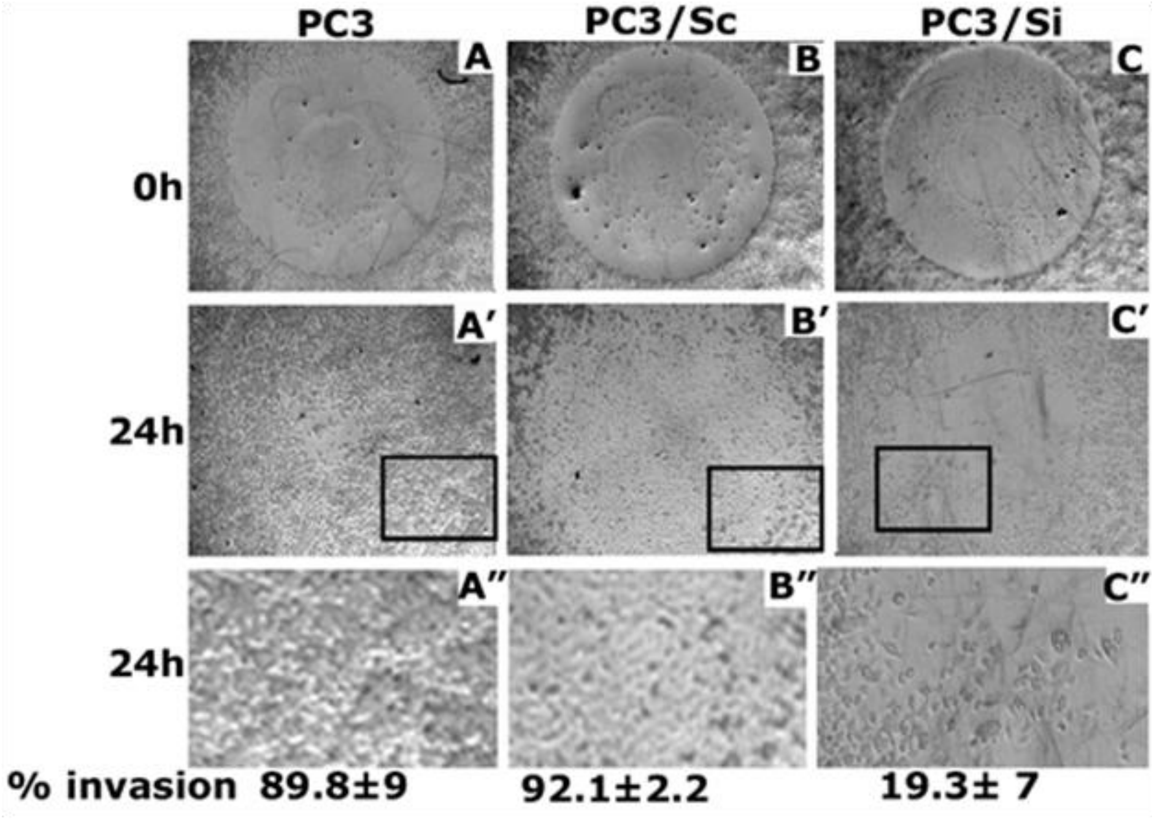

Figure S1: **Determination of migration/invasion property of indicted PC3 cells by the Oris cell invasion assay (3D assay).** Images were taken by use of an inverted Nikon microscope (Eclipse TE200) with 4X (A-C), 10X (A'-C') and 40X (A"-C") objectives. Representative images of pre-migration/invasion zones at 0h and migrated/invaded zones after 24h (A'-C') are shown. Invaded regions are shown at higher magnification in A"-C". Cells moved through the basement membrane extract (BME) (A'-C') and invaded into the BME (A" and B"). PC3 cells knock down of MMP9 displayed a significant decrease in migration/invasion (C' and C"). Cell migration/invasion into detection zone (towards center) was represented as percent invasion at the bottom of each panel. The results shown are representative of three independent experiments.

Figure S2

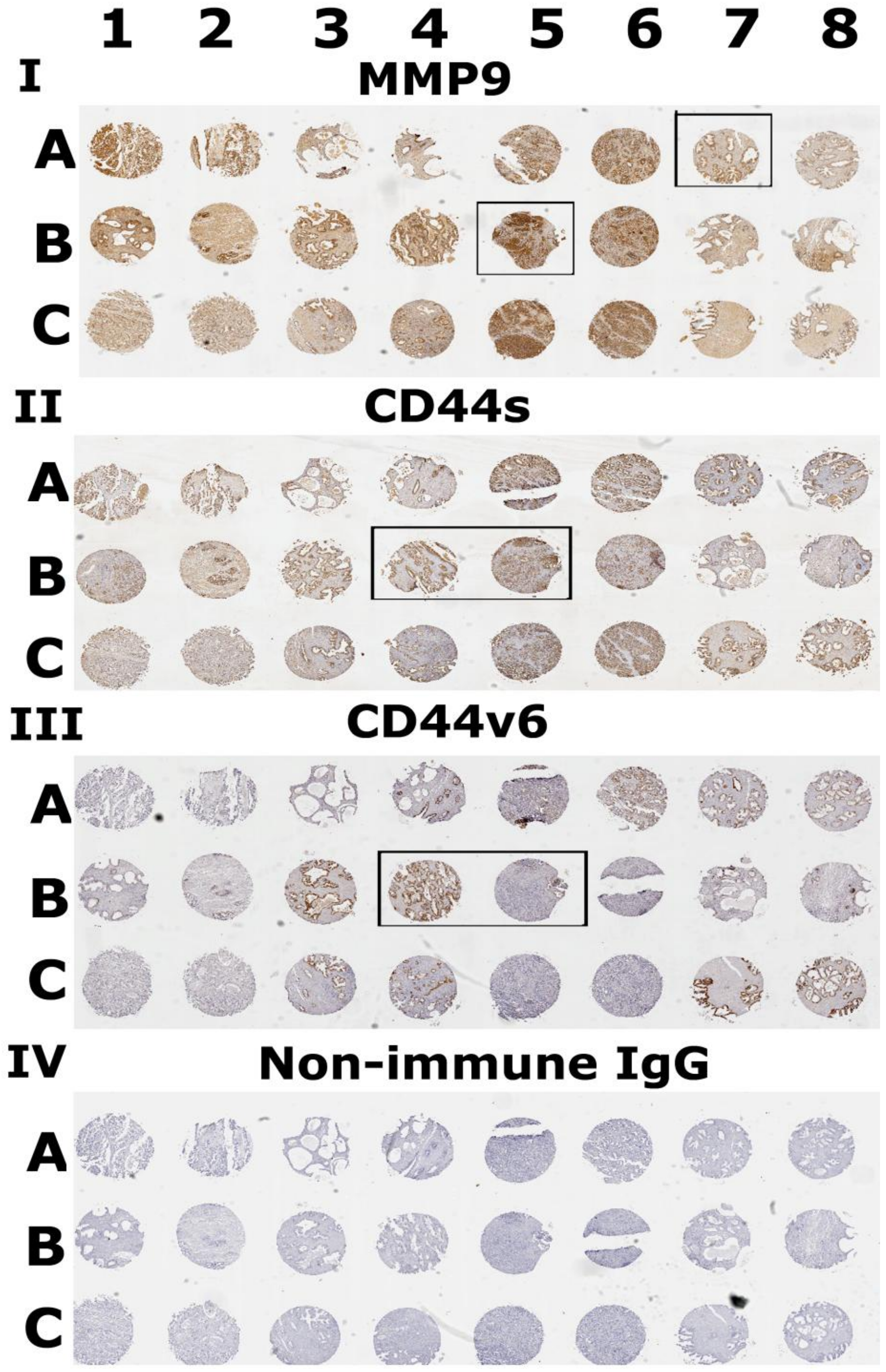

<u>Figure S2: Prostate cancer and normal tissue microarray (TMA).</u> Prostate adenocarcinoma at different stages and normal tissue cores (12 cases in duplicates) are stained with indicated antibody (I-III) and non-immune IgG (IV) are shown. The immunostained cores which are selected to show at higher magnification in Figure 8 of the main document are indicated by a rectangular field above. TMA containing 24 and 40cases were also used for this analysis. Relative distribution of indicated proteins in immunostained TMA sections were semi-quantitatively analyzed by two other investigators and provided as graph in the main document.

Figure S3

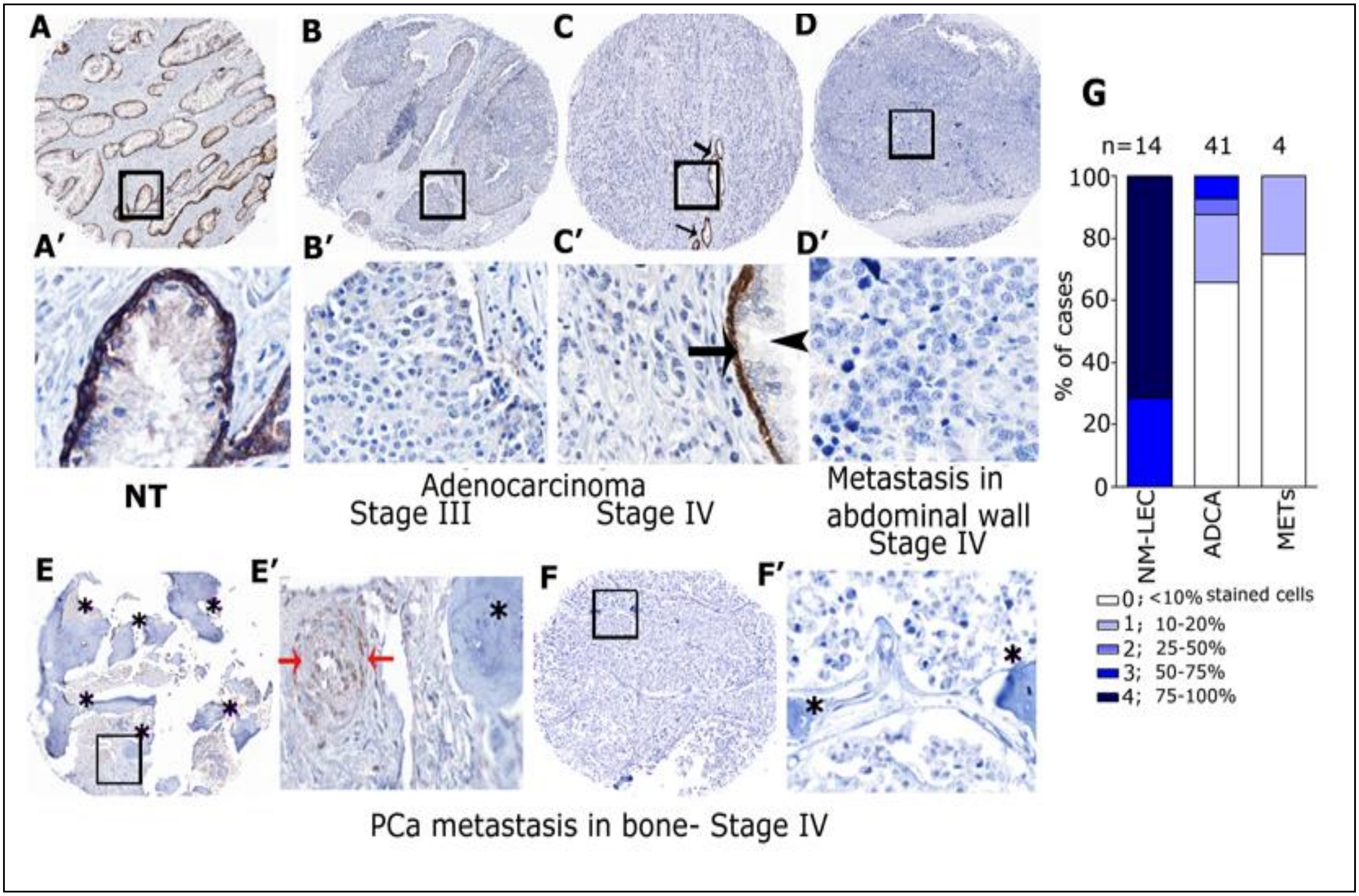
A
B
C
D
G
A′
B′
C′
D′
n=14
41
4
100
80
60
40
20
0
% of cases
NM-LEC
ADCA
METs
NT
Adenocarcinoma
Stage III
Stage IV
Metastasis in
abdominal wall
Stage IV
0; <10% stained cells
1; 10-20%
2; 25-50%
3; 50-75%
4; 75-100%
E
E′
F
F′
PCa metastasis in bone- Stage IV

Figure S3: Immunohistochemical detection of CD44v6 in normal prostatic tissue, prostatic adenocarcinoma (stage 3 and 4) and metastatic adenocarcinoma. **A-F and A'-F':** Prostate adenocarcinoma tissue microarray with duplicated cores of 36 cancer and three cases of metastasis to bone and one in abdominal wall was immunostained with an antibody to CD44v6. In the 36 cancer duplicate cores, 8 of which have matched normal adjacent tissue (Cat. No. PR956; BioMax, Inc). Representative tissue sections obtained from A-F': Immunohistochemical detection of CD44v6 in normal tissue (A and A'), adenocarcinoma at stage 3 and 4 (B, B', C and C') and metastatic cancer in abdominal wall (D and D') and bone (E, E', F and F') is shown. NT represents normal tissue. Magnification is X50 in A-F. Location of the high magnification (X200) regions shown in A'-F' is indicated by a rectangle field in A-F. **G:** Semi-quantitative analysis of distribution of CD44v6 in normal prostatic luminal epithelial cells in prostatic tissue (NM-LEC), prostatic adenocarcinoma (ADCA; stage 3 and 4) and metastatic adenocarcinoma in bone (METs). Expression of CD44v6 was measured semi-quantitatively via using the intensity of the immunoreactivity of CD44v6. It was calculated using the 0 to 4 tiered scale as shown in Figure S3G. The percentage was calculated based on the staining intensity of cells to CD44v6 protein in 4-tiered scale as shown in the figure G (see description below). 'n' indicates number of cores analyzed. The staining was repeated thrice with similar results.

RESULTS: Immunohistochemistry analysis for CD44v6 displayed negligible to no staining in the adenocarcinoma isolated at stage 3 (Figure S3B and B') and stage 4 (Figure S3C and C'), respectively. CD44v6 expression was stronger in normal tissue and observed in >90% of the luminal epithelial cells (A and A'). Note that intense staining of basolateral plasma membrane of luminal epithelial cells of normal tissue adjacent to prostate cancer at stage 4 (C and C'; indicated by arrows) matches with the staining in normal prostate tissue core (A and A'). Weak staining in the luminal epithelial cells is indicated by an arrow head. Prostatic cancer metastasized to abdominal wall (D) and bone (9E and F) showed weak reactivity for CD44v6 as well (indicated by red arrows). The relative distribution of CD44v6 in immunostained TMA sections containing 24 and 40 cases (in duplicates were semi-quantitatively analyzed by two investigators and provided in Figure S3G. While strong CD44v6 expression was observed in <5% of adenocarcinoma cases, 80-95% of normal cases show strong and very strong reactivity to CD44v6 in prostatic luminal epithelial cells (NM-LEC). We found that CD44v6 reactivity is consistently very low in adenocarcinoma at stage III to IV (<10% stained; ADCA) and in prostate cancer metastasis to bone (<5% stained; METS). Taken together, these observations imply that the switch in expression to CD44s from CD44v6 occur in metastatic cancer.
